# Microstructure and Wear Properties of Micro Arc Oxidation Ceramic Coatings

**DOI:** 10.3390/ma13040970

**Published:** 2020-02-21

**Authors:** Xiaoben Qi, Hailong Shang, Bingyang Ma, Rulin Zhang, Leyang Guo, Bo Su

**Affiliations:** School of Materials Engineering, Shanghai Dianji University, Shanghai 201306, China; qb85228@163.com (X.Q.); Maby@sdju.edu.cn (B.M.); Zhangrl@sdju.edu.cn (R.Z.); a15000372173@163.com (L.G.); bosu_sdju@126.com (B.S.)

**Keywords:** micro arc oxidation, electrical parameters, microstructure, wear resistant, ceramic coating

## Abstract

The interaction effect of micro arc oxidation (MAO) parameters on the microstructure and wear properties was investigated. The results showed that the electric current and oxidation time significantly influenced the thickness and grinding crack width of the ceramic coatings within the range of the selected parameters, and the interaction effect of the electrical parameters was not obvious. The surface morphology, cross-section morphology, and element distribution of the coatings were observed using scanning electron microscopy (SEM) with energy dispersive spectroscopy (EDS) and X-ray diffraction (XRD). The results showed that ceramic coatings with γ-Al_2_O_3_ and α-Al_2_O_3_ formed, which enhanced the coating performance. After that, the microhardness and wear resistance were tested. Under the optimal process, the microhardness of a coating section was up to 1200 HV_0.1_, and the friction coefficient was just 0.3. When wear occurred, the volcanic microstructures experienced extrusion and deformation, and then peeled off under shear stress, which led to the formation of a grinding crack. The main failure modes of the micro arc oxidation coatings were abrasive wear and spalling failure.

## 1. Introduction

Micro arc oxidation (MAO) as a novel technique has been widely used to deposit ceramic coatings on metal surfaces [1,2]. Owing to its high efficiency and environmentally friendly properties, this technology has gradually replaced traditional surface treatment processes. Wang Shaopeng [3] studied the morphology and wear resistance of the composite coatings formed on a TA2 substrate using micro arc oxidation. Qin Decai [4] researched multiphase ceramic coatings with a high hardness and wear resistance using the micro arc oxidation method. Leslaw Kyziol et al. [5] investigated the stress corrosion of micro arc oxidation coatings. Micro arc oxidation ceramic coatings with strong bonding and compact structures are naturally grown on the metal surface, a method resulting in excellent properties, such as a high micro hardness, good adhesion, wear resistance, and corrosion resistance. 

However, micro arc oxidation is a complex process that contains electrochemical and plasma-chemical reactions. Many factors affect the microstructure and properties of ceramic coatings. A lot of researchers have focused on the effects of electrolyte and electrical parameters on the microstructure and properties of coatings. Sang Sik Byeon [6] studied the effect of various nitrogen solutions on an oxide coating microstructure. Wei Song [7] investigated the influence of a stable solid electrolyte mixed with a cellulose additive on the tribological performance of micro arc oxidation coatings. In general, micro arc oxidation is conducted in an aqueous electrolyte, however, Alexander Sobolev [8] applied an alternative MAO ceramic coating to a Ti-6Al-4V alloy in molten nitrate salt at a temperature of 280 °C. Not only the electrolyte type, but also the temperature of the electrolyte had a great influence on the coating quality. Wang Xuefei [9] studied the characterization of micro arc oxidation coatings on an aluminum alloy under different electrolyte temperatures, and found that the thickness and growth rate of the films decreased with the increase in electrolyte temperature, which led to a poor corrosion behavior. The influence of the electrolyte composition on the coatings was also one of the focuses of the research. Nowadays, alkaline solutions are more widely used in the micro arc oxidation of aluminum and its alloys. More scientists are paying attention to the development of multi-system component mixed electrolytes, which makes the study of micro arc oxidation more complicated. V.S. Rudnev [10] studied the effect of borate and silicate aqueous electrolytes on wear resistant oxide coatings. Electrical parameters, such as the electrical current, power frequency, duty cycle, and oxidation time, play an important role in ceramic coating generation. In the micro arc oxidation process, the anion transport process changes with the change of the electrical parameters. Many studies have focused on the effect of single factors on the microstructure and mechanical properties of ceramic coatings. The influence of an anodizing pre-treatment in sulfuric acid was investigated in the plasma electrolytic oxidation of aluminum in a silicate electrolyte under a constant root mean square (RMS) current by E. Matykina [1]. Wang Ping et al. [11] studied the characterization of micro arc oxidation coatings on aluminum drill pipes at different current densities, and the results showed that the coatings thickened with the incremental increase of the current density. E. Matykina [12] investigated whether the intermediate layer is promoted by AC micro arc oxidation, which enables control of the microdischarge characteristics and surface overheating. The use of a bipolar pulse current could improve the coating deposition efficiency, and the performance of the prepared ceramic coating is also better. Beyond that, the effects of DC plasma electrolytic oxidation [13] and current pulse frequency [14] on ceramic coatings have also been analyzed. Cui Xuejun [15] analyzed the effect of the deposition time on the structure and anti-corrosion properties of micro arc oxidation coatings. N. Sakhnenko [16] studied the relationship between the electrolyte composition and applied current density. Although the power frequency has no significant effect on the film thickness, it has a great influence on the porosity and roughness. The duty cycle represents the percentage of the total power over the time in one cycle, which affects the time and energy of the continuous discharge during micro arc oxidation.

The electrical parameters changed the transport process of the anions on the metal surface, which made a difference to the morphology and performance of the ceramic coatings. It is necessary to study the interactions among the electrical parameters on the coating performance. In this paper, the interaction effect of the electric current, power frequency, duty cycle, and oxidation time on the microstructure and the wear properties of the ceramic coatings were investigated by orthogonal experiment design. The microstructure, microhardness, and wear resistance were tested, and the corresponding relationships among them were also analyzed.

## 2. Experimental Details

### 2.1. Substrate Preparation

The substrate of the double-sided, glossy aluminum foil with Ra = 0.43 μm used in this study was commercially pure aluminum, with the following chemical composition: Fe 0.35 wt.%, V 0.05 wt.%, Ti 0.03 wt.%, Mn 0.03 wt.%, Zn 0.05 wt.%, Mg 0.03 wt.%, Cu 0.05 wt.%, Si 0.25 wt.%, and Al balance. Al foil with a thickness of 0.1 mm was cut into 50-mm squares. Then, the samples were polished with 800-grit SiC abrasive paper and were ultrasonically washed with acetone, after which the surface roughness of the aluminum foil increased to 0.63 μm. The purpose of polishing was to remove the impurities and to improve the bonding strength.

### 2.2. Micro Arc Oxidation Process

Micro arc oxidation equipment (MODEL T-MAO-B20) was provided by Xi’An Tian’Ao New Materials Company (Xi’An, China), for which the maximum output voltage was 750 V, the rated current was 450 A, and the pulse count number was continuously adjustable from 20 to 2000 Hz. In this study, a pulsed current model was used. 

In the micro arc oxidation process, many parameters affected the quality of the ceramic coatings. If the electric current was too low (<4 A), the arc failed, and if electric current was too high (>9 A), the surface ablation was serious; when the oxidation time was too short (<10 min), the coating thickness was only 5–10 μm, and when the oxidation time was too long (>60 min), the coating thickness increased slowly and the surface quality reduced, owing to the heat concentration in the solution. Besides these two parameters, the power frequency and duty cycle affected the coating surface quality. Therefore, the interaction between the four parameters is worth studying.

In this study, orthogonal experiments were used for data analysis, and the parameters of MAO, such as the electric current, power frequency, duty cycle, and oxidation time, as orthogonal factors, are presented in Table 1. Table 2 shows the orthogonal design table L_8_ (2^7^), where A × B represents the interaction between the electric current and power frequency, A × C represents the interaction between the electric current and duty cycle, and B × C represents the interaction between the power frequency and duty cycle.

The silicate electrolyte was prepared by mixing 2 g/L KOH, 5 g/L Na_2_SiO_3_, 3 g/L Na_2_B_4_O_7_, and 3 g/L (NaPO_3_)_6_ in distilled water, and the solution temperature was kept below 40 °C using recirculation cooling water during the MAO process. 

### 2.3. Coating Microstructure and Wear Properties

After the MAO process, the samples were cleaned in acetone and then dried. The surface morphology and cross sections of the ceramic coatings were observed using a scanning electron microscope (SEM; S-4800, Hitachi, Tokyo, Japan). The elemental compositions of the ceramic coatings were investigated by SEM using an energy dispersive spectrometer (EDS; EDAX Appolo XP, Hitachi, Tokyo, Japan). The phase compositions of the coating surfaces were examined using an X-ray diffractometer (XRD; D/Max-255, Rigaku, Tokyo, Japan).

The microhardness of the ceramic coating sections was measured using a microhardness tester (HXS-1000A, Shanghai Optics Apparatus Ltd., Shanghai, China), with a load of 100 g and a loading time of 10 s.

The structure of the ceramic oxide coatings consists of a low-density porous upper layer, a high-density defectless working layer, and a transition layer, from which the external layer should be removed [17]. Therefore, before the wear experiments, the samples were polished again with 800-grit SiC abrasive paper with a surface roughness of 0.6 μm. The wear experiments of the coating surfaces were performed by a micromechanical testing machine. The grinding balls were made of E52100 chromium steel, of which the microhardness was about 63 HRC. The friction track length was 3 mm, the loading force was 5 N, and the friction cycle was 3 s. The friction time was set to 5 min. The average friction coefficients of the samples were recorded and the wear surface morphology was observed using a scanning electron microscope.

## 3. Results and Discussion

### 3.1. Interactive Orthogonal Test

**Figure 1:**
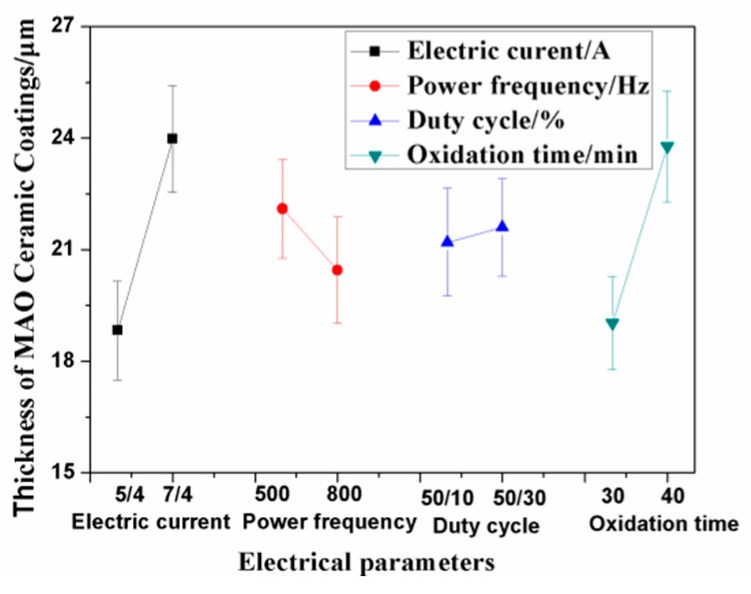
Relationship between the electrical parameters and coating thickness. MAO—micro arc oxidation.

As shown in Table 2 for the orthogonal test results, the thickness of the ceramic coatings and the surface grinding crack width were regarded as the investigation targets, which were affected by the electrical parameters, such as the electric current, power frequency, duty cycle, and oxidation time. The experimental data and calculation results from the L_8_ (2^7^) orthogonal array design are also given in Table 2. Combined with the range analysis, the interaction effect of the electrical parameters on the coating thickness and grinding crack width were obtained, which are shown in Table 2. Therein, the Mean1/T value equaled the mean value from T1 to T4, which was influenced by the changes in Sample Tests 1, 2, 3, and 4. The values of Mean1/T and Mean2/T show the ranking of significance of each of the MAO parameters on the coating thickness and grinding crack width. Within the range of the selected parameters, electric current had the greatest influence on the thickness of the ceramic coatings, followed by the effect of oxidation time, shown in Table 2. The increase in electric current directly affected the temperature of the reaction zone. The higher the electric current, the greater the energy in the reaction area, which promoted coating growth. The longer the oxidation time, the thicker the ceramic coatings. Other influencing factors, such as the duty cycle and power frequency, had little effect on the coating thickness. However, the two factors affected the porosity of the ceramic coatings [18]. The interaction effect of the electric current density and the duty cycle, and the power frequency and duty cycle were not significant within the range of the selected parameters. Figure 1 shows the relationship between the electrical parameters and the thickness of the ceramic coatings. Combining the results of Table 2 and Figure 1, parameter combinations, such as A2 (7/4A), B1 (500Hz), C1 (50/10%), and D2 (40 min), could be calculated and determined as the optimal levels of factors A, B, C, and D, respectively. 

### 3.2. Microstructure and Phase Analysis of the Ceramic Coating

**Figure 2:**
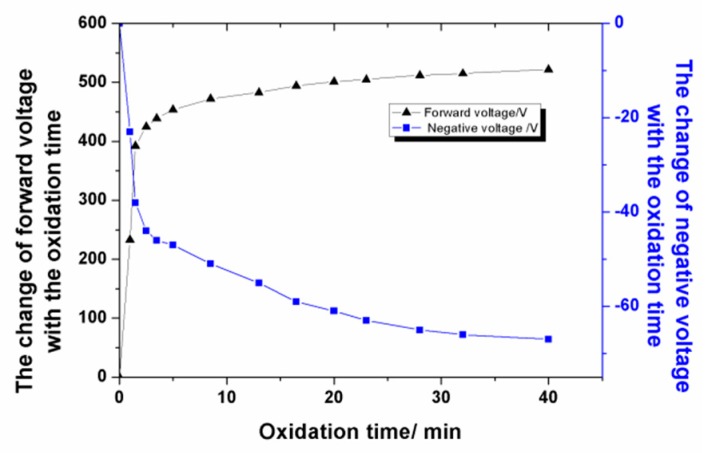
The change of voltage with the oxidation time.

The change in voltage over the oxidation time under the optimum process parameters was analyzed. At the beginning of the MAO experiment, as shown in Figure 2, the voltage value increased rapidly with the oxidation time, and a large number of bubbles existed on both surfaces of the Al foil. After about 2.5 min, the positive voltage value increased to 450 V rapidly, with a negative voltage of −44 V, and countless sparkles moved rapidly on the Al surface. The final positive voltage value increased up to 500 V, with a negative voltage of −67 V. 

**Figure 3:**
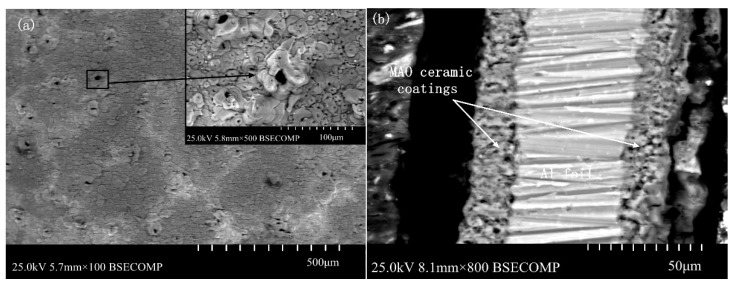
The surface morphology and cross section of the MAO ceramic coatings: (**a**) surface morphology and (**b**) cross section.

Figure 3 shows the surface morphology and cross section of the MAO ceramic coatings. As shown in Figure 3a, many small craters were distributed on the coating surface, and some large holes appeared in some areas, which were formed by the accumulation of molten materials. In addition, cracks existed in the surrounding area, connecting the surrounding holes. As shown in Figure 3a, a large amount of sprayed molten materials, microporous structures, and micro-cracks were maintained on the coating surface. These unevenly distributed and irregularly sized holes are channels for the medium particles in the MAO process. 

**Figure 4:**
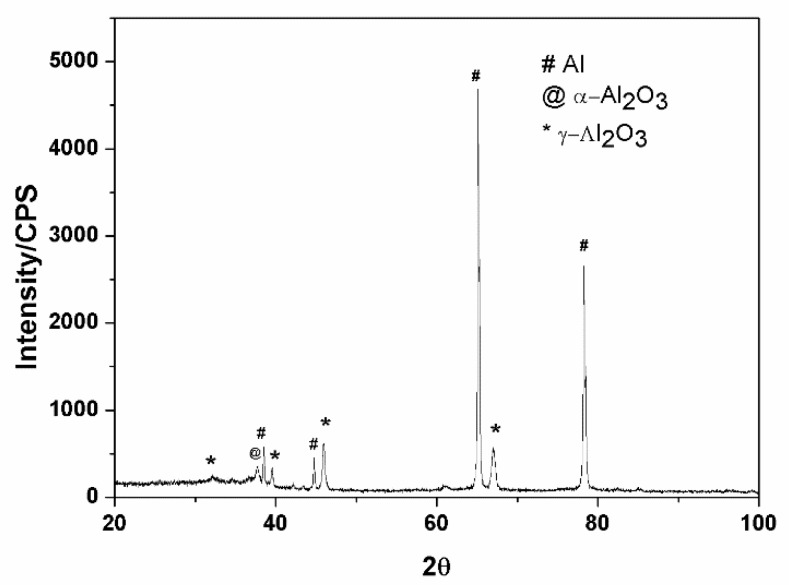
XRD analysis of the ceramic coatings.

The XRD pattern analysis of the ceramic coatings is presented in Figure 4. The results show that the main component of the ceramic coatings contained α-Al_2_O_3_, γ-Al_2_O_3_, and Al phase. The existence of an Al peak showed that the X-ray passed through the ceramic coatings, and the Al substrate was characterized. The existence of the α-Al_2_O_3_ and γ-Al_2_O_3_ phase illustrated that they formed among many molten pools at the moment of discharge, owing to the instant high temperature; these two kinds of phases have been verified to improve the hardness of the coatings [19,20]. The difference in the cooling rate of the molten alumina in the micro arc zone resulted in the difference in the α-Al_2_O_3_ and γ-Al_2_O_3_ content between the external and internal layers of the coatings [21]. Some researchers [22,23] reported a porous outer-layer region consisting predominantly of low temperature γ-Al_2_O_3_, and a dense internal region consisting predominantly of high temperature α-Al_2_O_3_. If the content of the α-Al_2_O_3_ phase in the compact layer is over 50%, the compact layer has a very high microhardness and excellent wear resistant performance [24].

**Figure 5:**
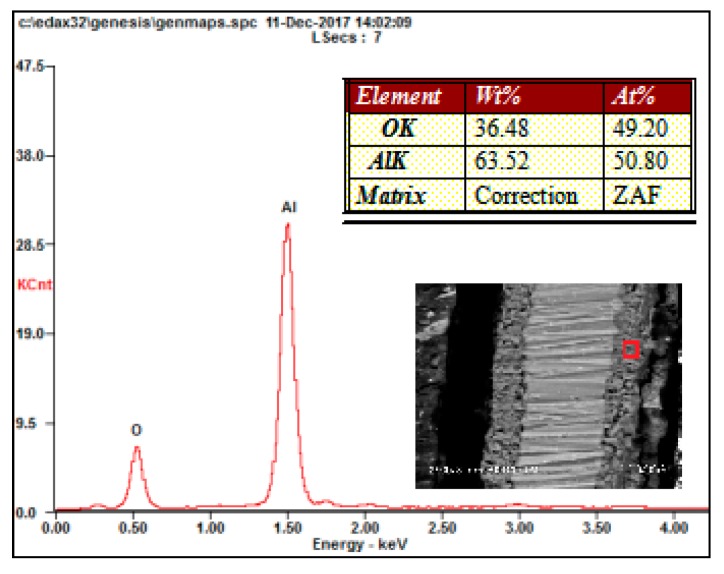
EDS analysis of the ceramic coatings.

As shown in Figure 3b, the thickness of the ceramic coatings was uniform, and the MAO ceramic coatings were well bonded to the Al substrate. Only some poles existed inside the ceramic coatings. The elemental composition of the ceramic coatings was investigated by SEM with an energy dispersive spectrometer, and the results are shown in Figure 5. As shown in these results, elements of Al and O were detected in the coatings. Because the oxygen content was not accurately measured by SEM, the atomic ratio of Al and O was not exactly 2:3. However, combined with the XRD results, the formation of Al_2_O_3_ can be known.

### 3.3. Wear Properties of Ceramic Coating

**Figure 6:**
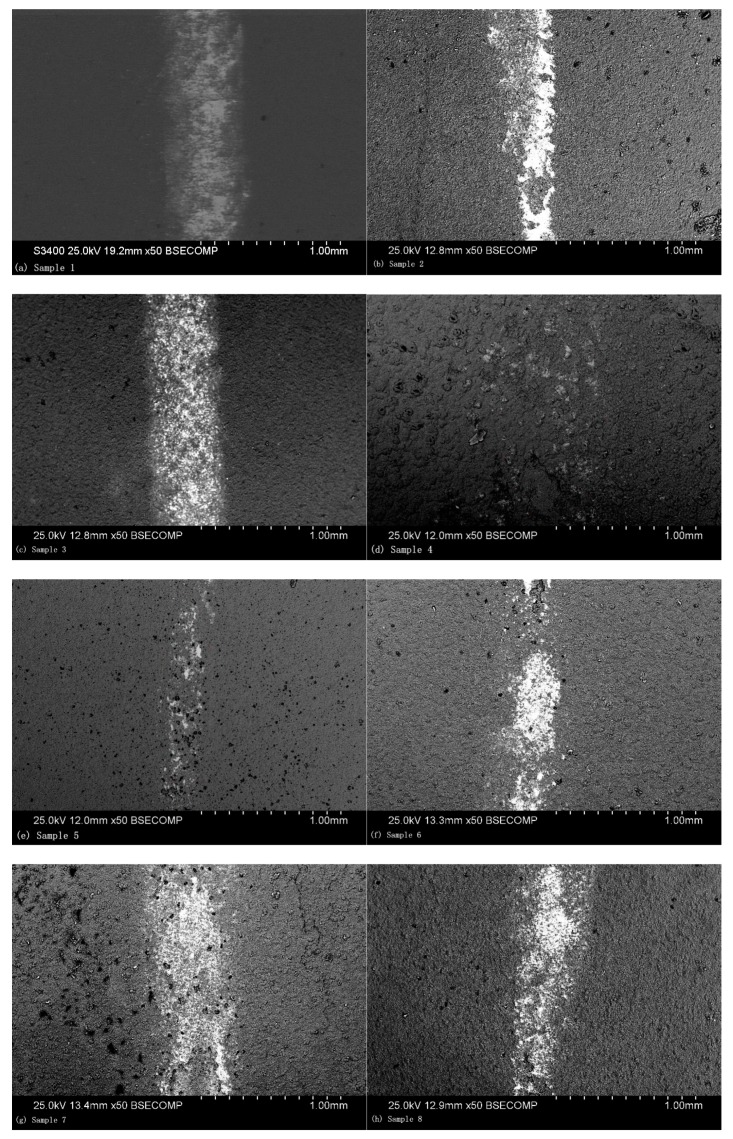
SEM observation of the grinding crack morphology: (**a**) Sample 1, (**b**) Sample 2, (**c**) Sample 3, (**d**) Sample 4, (**e**) Sample 5, (**f**) Sample 6, (**g**) Sample 7, and (**h**) Sample 8.

The grinding crack morphologies of the coatings were observed, as shown in Figure 6, which presents the grinding crack morphology of orthogonal Test Samples 1–8. It is indicated in this figure that shallow grooves along the sliding direction were present. The ceramic coating surface exhibited much more raised microstructures, like volcanos, which were formed by the solidification of the ceramic coatings. When wear happened, the grinding ball was first in contact with the volcanic structures of the coating surface, which experienced extrusion and deformation, and then broke up into fine particles under shear stress and were distributed on the friction surface, after which the grinding crack formed.

**Figure 7:**
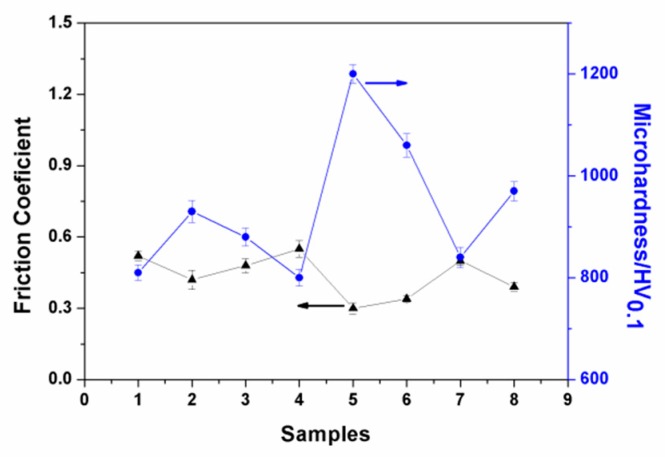
Friction coefficient and microhardness of the ceramic coatings.

Different parameters resulted in different grinding crack widths. As presented in Figure 6e, the grinding crack width was the most narrow, which is in accordance with the results of the orthogonal test. As shown in Figure 6, there were hardly any volcanic structures seen on the worn surface, which indicates that the abrasive particles were filling in the region [25]. The average friction coefficient of the ceramic coatings at a steady state were also recorded, for which the results are presented in Figure 7. As can be seen in this figure, the experiment results showed that the friction coefficients at steady state were between approximately 0.3 and 0.6. Among them, the friction coefficient of Sample 5 was the smallest, for which the wear marks were also the narrowest. In addition, the microhardness of the coating sections was tested. As given in Figure 7, it can be seen that there was a good correspondence between the microhardness and friction coefficient. The higher the microhardness of the coatings, the better the wear resistance and the lower the friction coefficient.

**Figure 8:**
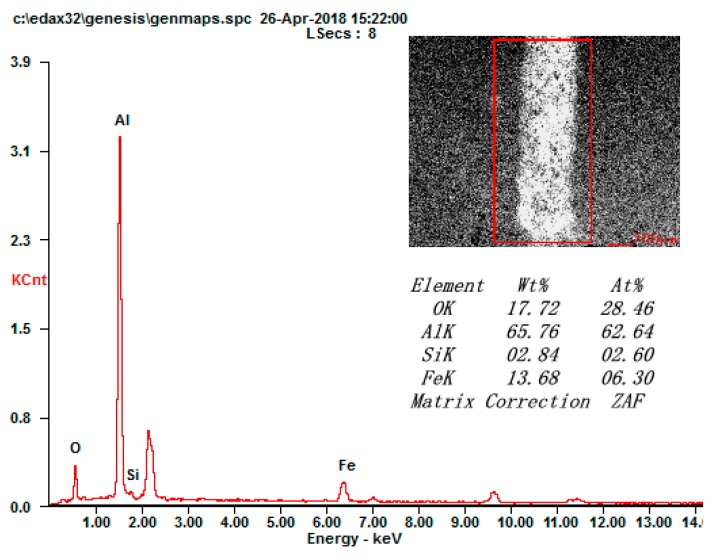
EDS analysis of the grinding crack surface.

The components of the grinding cracks were further analyzed, which are shown in Figure 8. The existence of element Fe on the grinding crack surface illustrates that the materials of the grinding balls adhered to the coating surface when wear occurred. The formation of the α-Al_2_O_3_ and γ- Al_2_O_3_ phases from the micro arc oxidation process meant that the ceramic coating was composed of a compact layer with α-Al_2_O_3_ and a loose layer with γ-Al_2_O_3_. The improvement of the surface properties is mainly attributed to the phase of α-Al_2_O_3_ [26,27]. Thus, the abrasive wear and spalling failure were the main wear mechanisms of the micro arc oxidation coatings.

**Figure 9:**
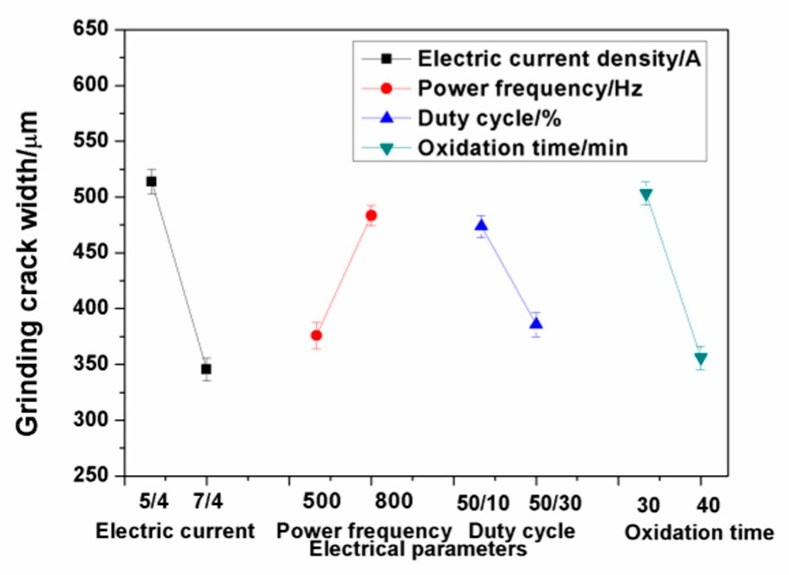
Relationship between the electrical parameters and grinding crack width.

Figure 9 shows the relationship between the electrical parameters and grinding crack width. The results show that the main influence of the electrical parameters on the grinding crack width were the electric current and oxidation time. The grinding crack width of the ceramic coatings decreased with the increase of the electric current and oxidation time within the range of the selected parameters. The thickness of the compact layer in the ceramic coatings increased when the electric current increased, which is beneficial to the improvement of the wear resistance. 

## 4. Conclusions

In the present work, ceramic coatings were prepared on Al foil using the micro arc oxidation method. The interaction effects of the electrical parameters on the thickness and grinding crack width of the coatings were investigated. Coating microstructures like volcanos formed on the Al foil surface during discharge. In the range of the selected parameters, the electric current and oxidation time had the greatest influence on the coating thickness and grinding crack width. The major compositions of ceramic coatings contained γ-Al_2_O_3_ and α-Al_2_O_3_, which were beneficial to the coating performance. When wear occurred, the microstructure like volcanos first made contact with the grinding ball, and then peeled off under shear stress. The main failure mode of the coatings were abrasive wear and spalling failure.

## Figures and Tables

**Table 1:** Experimental parameters of the orthogonal test.

Level	Micro arc oxidation parameters			
	(A) Current *I* (Positive/Negative) (A)	(B) Power frequency *F* (Hz)	(C) Duty cycle (%)	(D) Oxidation time (min)
1	5/4	500	50/10	30
2	7/4	800	50/30	40

**Table 2:** Experimental results and range analysis of the orthogonal test.

Sample	A (A)	B (Hz)	A × B	C (%)	A × C	B × C	D (min)	T (thickness/μm)	W (worn width/μm)
1	1(5/4)	1(500)	1	1(50/10)	1	1	1(30)	18.1 ± 1.3	572 ± 10
2	1	1	1	2(50/30)	2	2	2(40)	21.3 ± 1.4	376 ± 12
3	1	2(800)	2	1	1	2	2	20.5 ± 1.6	519 ± 9
4	1	2	2	2	2	1	1	15.4 ± 1.0	588 ± 11
5	2(7/4)	1	2	1	2	1	2	26.8 ± 1.4	253 ± 13
6	2	1	2	2	1	2	1	23.2 ± 1.2	302 ± 12
7	2	2	1	1	2	2	1	19.4 ± 1.5	551 ± 8
8	2	2	1	2	1	1	2	26.5 ± 1.6	276 ± 8
Mean1/T	18.83	22.10	21.33	21.20	22.08	21.70	19.025		
Mean 2/T	23.98	20.45	21.48	21.60	20.73	21.10	23.775		
Range T	5.15	1.65	0.15	0.40	1.35	0.60	4.75		
Mean1/W	513.75	375.75	443.75	473.75	417.25	422.25	503.25		
Mean2/W	345.50	483.50	415.50	385.50	442.00	437.00	356.00		
Range W	168.25	107.75	28.25	88.25	24.75	14.75	147.25		
The influence of the parameters on the coating thickness: A > D > B > A × C > B × C > C > A × B									
The influence of the parameters on the grinding crack width: A > D > B > C > A × B > A × C > B× C									
